# Inpatient Opioid Use Disorder and Social Determinants of Health: A Nationwide Analysis of the National Inpatient Sample (2012-2014 and 2016-2017)

**DOI:** 10.7759/cureus.11311

**Published:** 2020-11-03

**Authors:** Saanie Sulley, Memory Ndanga

**Affiliations:** 1 Health & Biomedical Informatics, National Healthy Start Association, Washington DC, USA; 2 Health Information Management, Rutgers University, Piscataway, USA

**Keywords:** social determinants of health, opioid use disorder, housing, unemployment, literacy, healthcare access, psychosocial stress, inpatient admissions, healthcare disparities, health inequities

## Abstract

Objective

To evaluate the trends and relationship of inpatient presentations of Social Determinant of Health (SDOH) with superimposed Opioid Use Disorder (OUD), comparing 2012-2014 (ICD-9) and 2016-2017(ICD-10).

Methods

We used the Nationwide Inpatient Sample (NIS) from the Healthcare Cost and Utilization Project (HCUP). We identified OUD among patients with any record of SDOH as the primary or secondary diagnosis using the International Classification of Diseases (ICD)-9/10 codes. A weighted SDOH sample size of 3,002,558 (2.8%) and 1,254,899(1.8%) was included for 2012-2014 and 2016-2017, respectively. The main predictors include census division, race, gender, and covariates, including age, income, disposition, payer, rural-urban classification, and combined SDOH indicator, which was used as a control variable in the regression analysis. The study provides a descriptive analysis of the social determinant of health in relation to OUD. We also evaluated the rate of the presentation by age group and race.

Results

A sample of 367,960 (12%) and 153,535 (12%) OUD presentations with SDOH indicators were identified for 2012-2014 and 2016-2017, respectively. An increase in housing difficulties between 2016-2017 (45%) as compared to 2012-2014 (20%) was observed. A statistically significant higher odds of presentation among black and other races were observed. There was significant variance in the presentation by region with Middle Atlantic with OR-2.04, 95 C. I (1.98, 2.10) and East North Central with OR-1.94, 95 C. I (1.89, 1.99). Higher admissions were observed for both 2012-2014 and 2016-2017 among patients without any payment method classified as no charge. Statistically significant relationships (p<0.05) were also observed among low income <$54,000 OR-1.01, 95 C. I (0.99, 1.04), Housing OR-1.49, 95 C. I (1.47, 1.52), Primary Support OR-0.93, 95 C. I (0.90, 0.95), Employment OR-1.37, 95 C. I (1.35, 1.39), Psychosocial OR-1.63, 95 C. I (1.59, 1.67), Age group 25-34 OR-15.64, 95 C. I (14.20, 17.22), 35-44 OR-29.07, 95 C. I (26.45, 31.95) in both 2012-2014 and 2016-2017.

Conclusion

SDOH has a direct impact on inpatient OUD presentations. Socio-economic disparities exist in all census regions, race, sex, and rural-urban demographics. Interventions aimed at reducing the incidence and risk of OUD should focus on specific local dynamics using a multidisciplinary, data-driven quality improvement (QI) approach to address the root cause of presentations effectively. A community-based approach to addressing SDOH through collaboration with care providers could play a substantial role in decreasing length of stay (LOS), cost, and potential readmission among these populations.

## Introduction

The United States has seen a significant increase in prescription drug misuse that has led to the opioid crisis. Opioid Use Disorder (OUD) impacts the lives of over 2.5 million Americans from all walks of life [1]. In 2013, an estimated 78 billion dollars was spent on prescription-related opioid-related disorders, and one-third was geared toward physical and behavioral health services [2]. Several factors contribute to the ongoing epidemic, including social and economic indicators in various communities across the country [3-4]. OUD's economic impacts not only affected individual users but substantially contributed to high healthcare costs, criminal justice, and lost workplace productivity [5]. As OUD-related expenditures continue to increase, there is a need to understand the socioeconomic factors associated with OUD hospital presentations. Understanding the socioeconomic factors related to opioid use will facilitate targeted therapy and intervention to combat the growing epidemic. Although prior studies have examined social and economic factors, they are either based on self-reported responses or focused on specific opioid populations and regions, which might not be nationally representative. Furthermore, the drastic increase in opioid use and hospitalized patients calls for constant review of the socioeconomic factors.

An examination of the existing literature shows that socioeconomic factors underlie OUD presentations. Socioeconomic barriers do not only predispose people to use drugs, but health disparities have also been noted in minority groups who are opioid users [6-7]. Studies have shown that delayed hospital presentation has an impact on the outcomes of diverse medical issues. Social factors contribute to the delay in seeking care for several medical illnesses, including those requiring acute treatments [8-9]. Moreover, they have also been associated with a higher risk of hospitalization, mortality, and more extended stay [10]. Socioeconomic status is essential, as we look at OUD to reflect cause and effects [11]. Hence the dire need to understand rural and urban socioeconomic factors about varied rural-urban OUD presentations [12-13]. This study aims to analyze the relationship between OUD and socioeconomic factors in the United States between 2012-2014 and 2016-2017.

## Materials and methods

This retrospective analysis was performed using the Healthcare Cost and Utilization Project's (HCUP's) Nationwide Inpatient Sample (NIS) data. The Agency for Healthcare Research and Quality (AHRQ) sponsors the HCUP databases that categorize hospital utilization trends and cost across the United States. We weighted the estimated samples to reduce the margin of error and to provide generalizable estimates based on data availability for the selected years. The NIS data source protects individual patients' privacy, and all hospital or provider identifiers are de-identified. 2015 data were not considered for this study because International Classification of Diseases (ICD) 10 was introduced in October 2015; therefore, the codes coding would have varied in the last quarter.

Study sample

ICD-9/10 identified the inclusion criteria for both SDOH and OUD presentation in this study for 2010-2014 and ICD 10 for 2016-2017. The list of codes used to identify SDOH and OUD are available in Appendix 1. Social factors impacting health include occupational, housing, unemployment, family, psychosocial, and healthcare access difficulties among U.S. hospitalizations. A weighted sample size of 3,002,558 (2.8%) and 1,254,899(1.8%) for 2012-2014 and 2016-2017, respectively. The predictors include census division, race, gender, and covariates, including age, income, disposition, payer, rural-urban classification, and combined SDOH indicator used as a control variable in the regression analysis. 

Analysis

We used the Statistical Package for the Social Science (SPSS) version 23.0 (IBM Corp., Armonk, NY) to conduct a retrospective analysis of the HCUP-NIS database from 2010-2014 and 2016-2017. Weighted descriptive statistics were used to summarize the results and rate of OUD presentations by variables of interest. We utilized the chi-squared test and multivariate logistic regression to evaluate the relationship and associations between the variables. We used logistic regression to assess the effects of SDOH factors and other variables of interest on the dependent variable (OUD presentation). We applied the discharge weight, which was given in the NIS database, to attain the inpatient data's national representation on all analyses. We used Power Bi (2019; Microsoft Corporation, Redmond, WA)) to calculate the rates and graph the statistics in this study.

## Results

**Figure 1:**
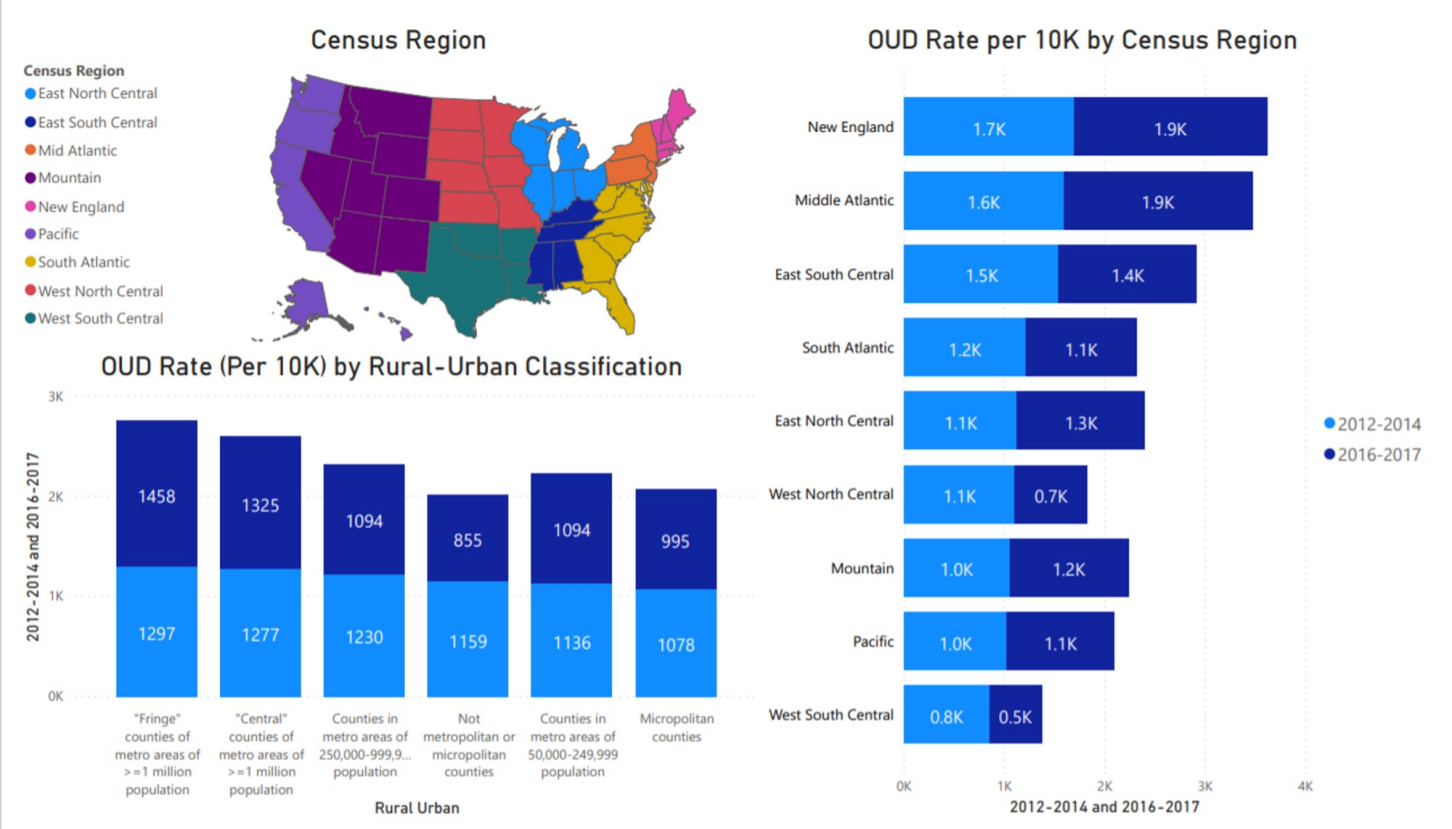
Overview of OUD presentations by region OUD: Opioid Use Disorder

The results in this study stratified by census regions help obtain a better understanding of the specific impact of these socioeconomic factors on the likelihood of OUD. Figure 1 provides an overview of OUD presentations by region. It shows a general rate increase in most regions except East South Central, South Atlantic, West North Central, and West South Central. Large counties and urban centers mostly saw an increase in rate, with a minor decrease in rural and non-urban areas across the country. The relationships were statistically significant, with p<0.001 as shown in Appendix 2.

Age and gender

**Figure 2:**
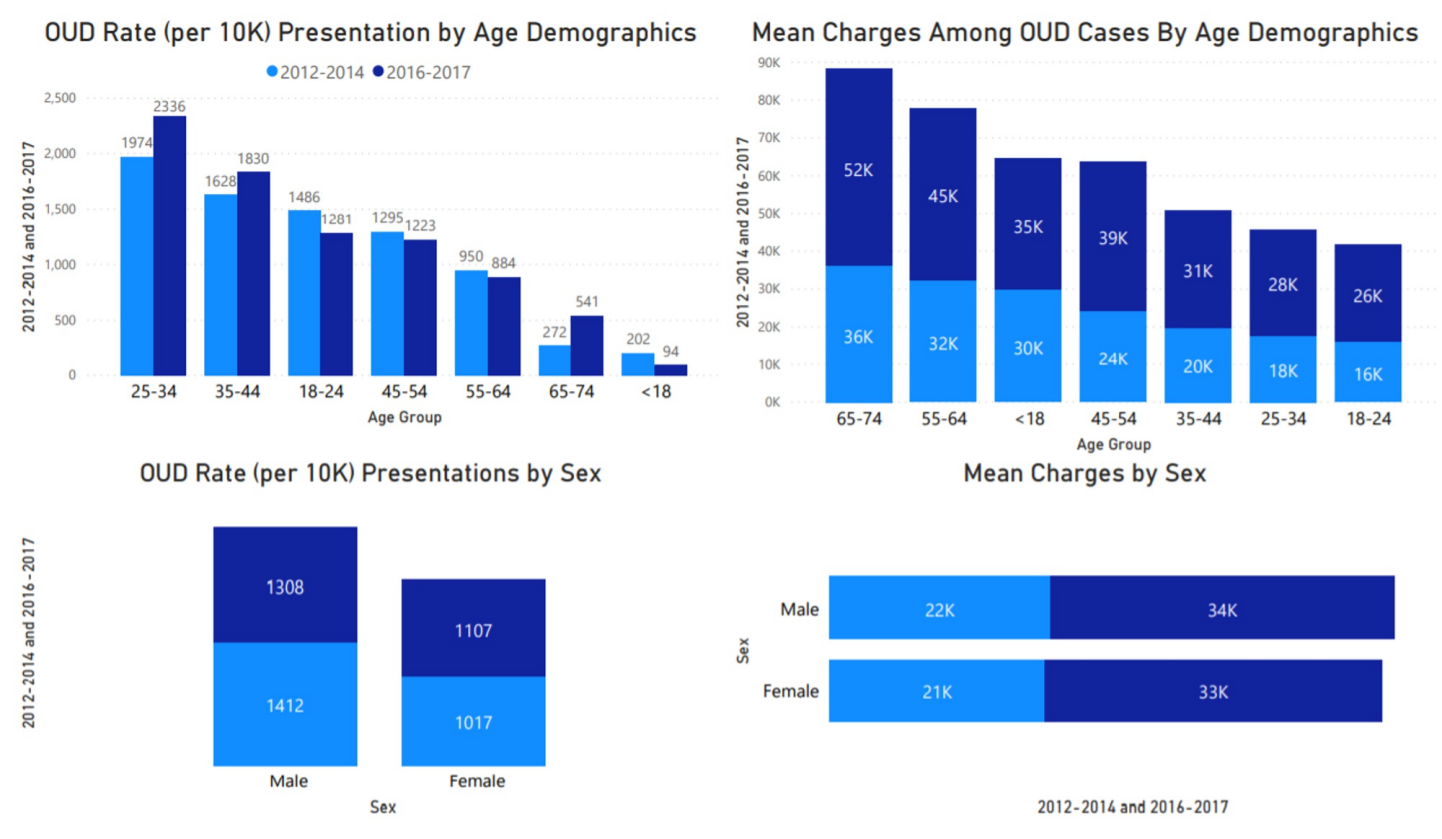
OUD presentation by age demographics, sex, and mean charges OUD: Opioid Use Disorder

Figure 2 shows age and sex demographics by OUD inpatient presentations. The age demographics for this population varied, with ages 25-34, 35-44, and 65-74 showing an increase in presentations in all regions across the country. Mean charges were higher for the 65-74 and 55-64 age groups, with high, increasing care costs for all age groups. Rate by sex shows a reduction in male presentation as compared to an increased female presentation. The cost of care also increased for both sexes by about $12,000 for the period included in this study. Length of stay (LOS) was higher among individuals <18 and 65-74, with an average of 15- and eight-days, respectively.

Race, income, and payer

**Figure 3:**
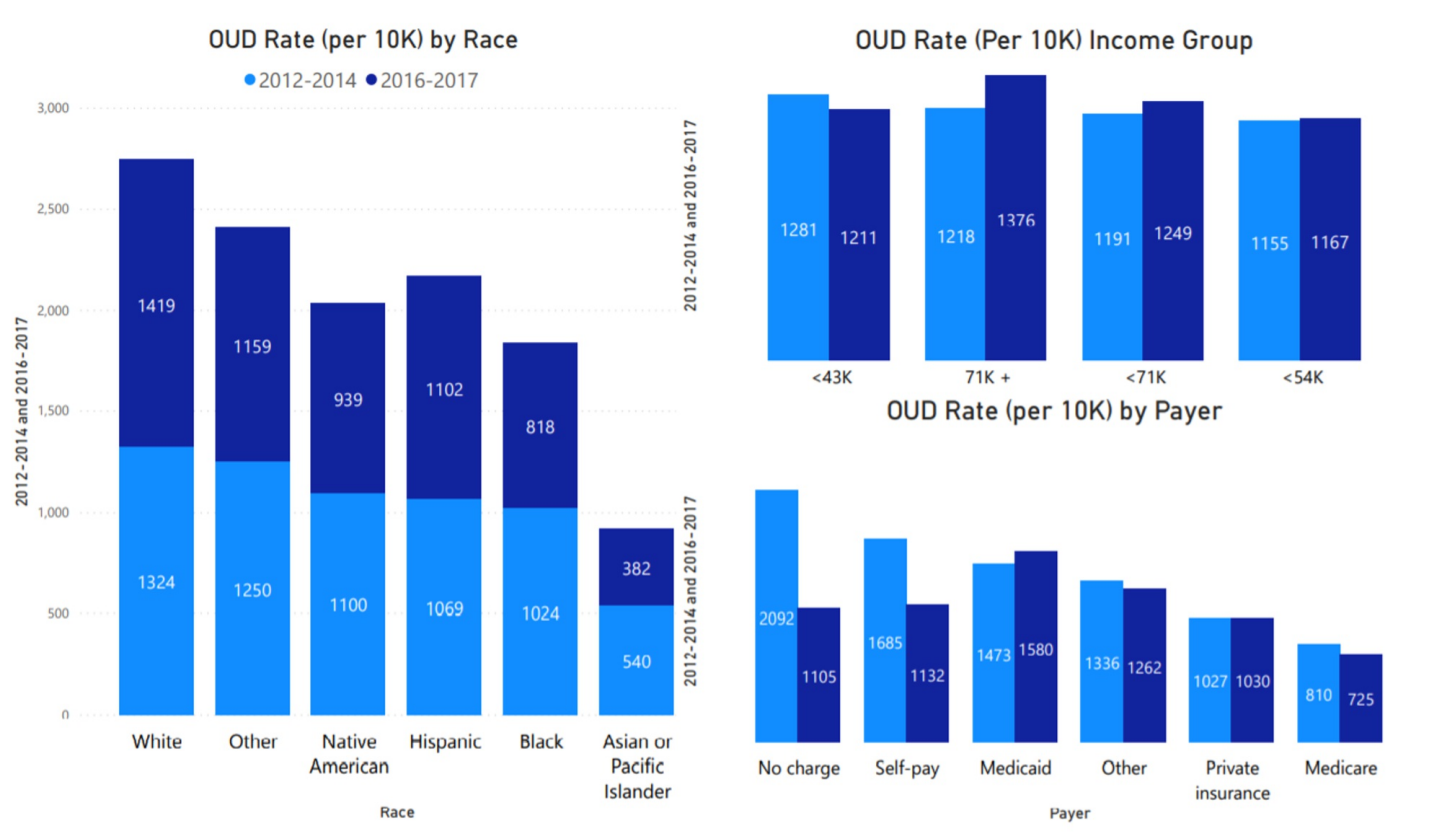
OUD presentation by race, income, and payer OUD: Opioid Use Disorder

As shown in Figure 3, racial demographics vary with white and other race classifications, with the highest presentation rate compared to blacks, Asians, or Pacific Islanders. There is a rate increase among white and Hispanic populations. Among income stratification, there is a rate increase among the highest income group in all regions. Medicaid rate among the population saw an increase (107) as compared to other payer demographics. All these mentioned relationships to OUD presentations were statistically significant (p<0.001). The racial demographic in overall OUD presentation by region shows an interesting makeup, with rate increase among the white and Hispanic populations across the country. The second and third highest presentation rates were among the other and Native American populations. Mean charges were higher among other, Hispanic & Asian, or Pacific Islanders. LOS was higher among Native American, other, and Asian or Pacific Islanders, with eight, seven, and seven days.

Employment status and housing difficulties

**Figure 4:**
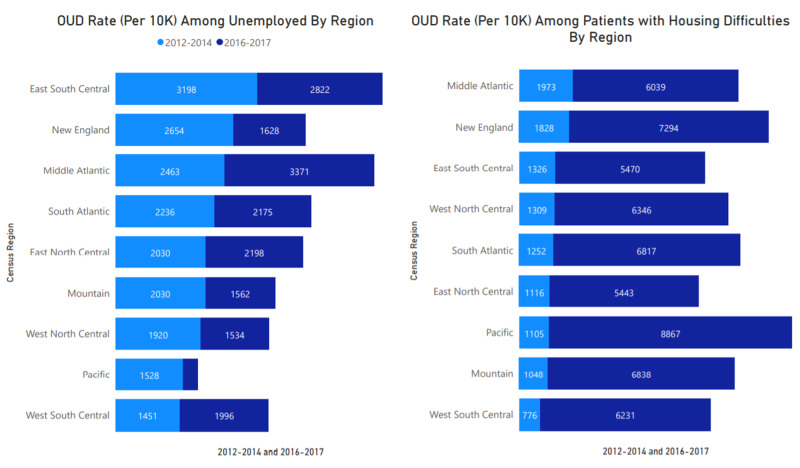
OUD rate among populations with unemployment and housing difficulties OUD: Opioid Use Disorder

As shown in Figure 4, the rate of unemployment among OUD presentations increased in the Middle Atlantic, East North Central, and West South Central regions. Minor reductions can also be observed in other areas across the country. The variance in rate could be related to the adoption and understanding of new ICD-10 codes on each social determinant of health and OUD classifications. Even though there seems to be an overall reduction in the presentation in some areas, there is a significant relationship (p<0.001) between unemployment and other employment difficulties and OUD presentations as shown in Appendix 2. Between 2012 and 2014, the difference between OUD combined with housing difficulties and those with only OUD presentation was 8.5-6 times higher throughout the United States. These rates were higher in the West North Central region (Missouri, North Dakota, South Dakota, Nebraska, Kansas, Minnesota, Iowa) followed by the West South Central region (Oklahoma, Texas, Arkansas, Louisiana) and the Middle Atlantic Region (New York, Pennsylvania, New Jersey) with high urban centers. The presentation rate was higher in the New England, Middle Atlantic, West South Central, East North Central. The figure also shows a significant association between housing and OUD presentation across regions in the United States. Higher rates between 2016 and 2017 may be associated with better data collection and the stratification of SDOH indicators in ICD-10 than ICD-9.

Family and psychosocial difficulties

**Figure 5:**
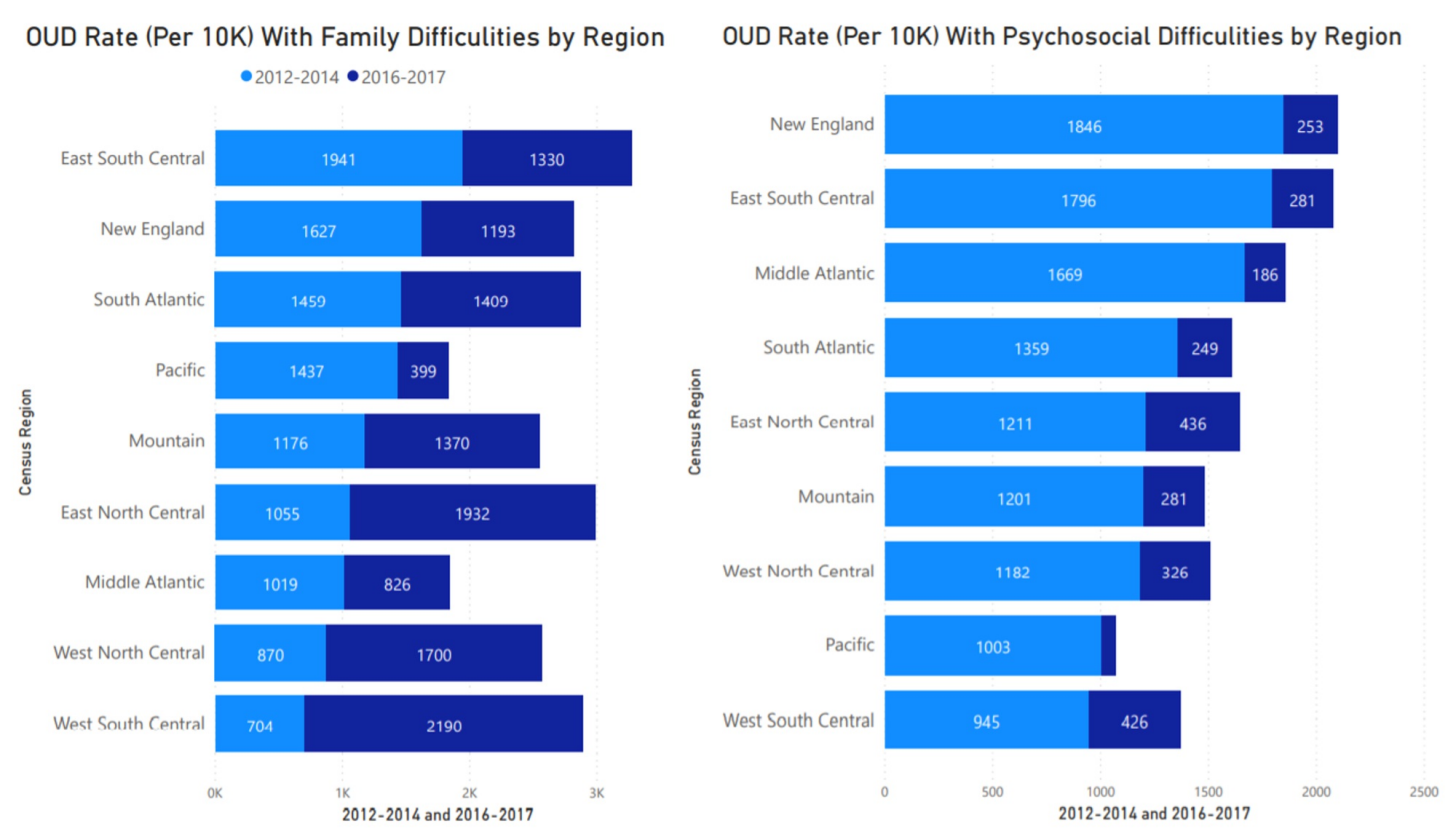
OUD rate among populations with family and psychosocial difficulties OUD: Opioid Use Disorder

Regional OUD presentation dynamics differ, as observed in Figure 5. East South Central, South Atlantic, Pacific, West South Central, Mountain, New England, West North Central, East North Central, and Middle Atlantic show higher OUD rates between 2012 and 2014. West South Central (Oklahoma, Texas, Arkansas, Louisiana) and East North Central (Wisconsin, Michigan, Illinois, Indiana, Ohio) show a high rate variance increase from 2012-2014 to 2016-2017. Rate reduction can also be observed in the New England, East South Central, Pacific, and Middle Atlantic regions. Among the general inpatient population, patients were 11 times more likely to present with OUD when they have some classification of psychosocial factors in the East North Central (Wisconsin, Michigan, Illinois, Indiana, Ohio) and West South Central (Oklahoma, Texas, Arkansas, Louisiana) regions between 2012 and 2014. The Pacific region (Alaska, Washington, Oregon, California, Hawaii) had the lowest OUD presentation likelihood of five times comparing those with and without documented psychosocial difficulties between 2012-2014 and 2016-2017. The significant variance in presentation rates may be associated with several factors, including potential coding effectiveness.

Healthcare access and education

**Figure 6:**
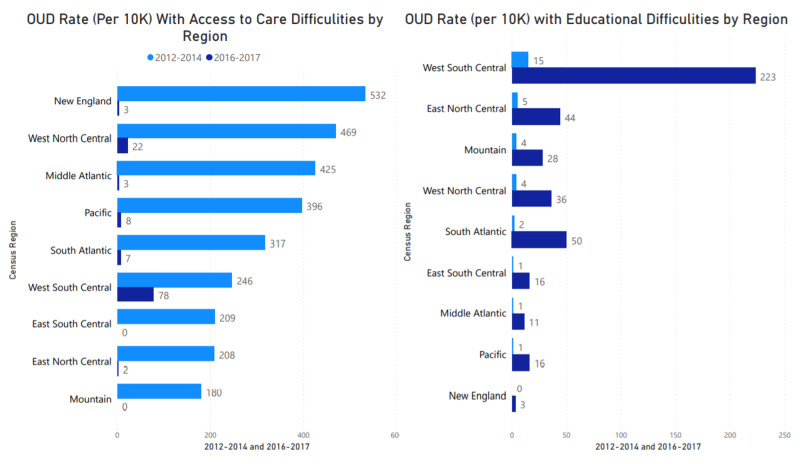
OUD rate among populations with access to healthcare and educational difficulties OUD: Opioid Use Disorder

The rate of OUD presentations (2012-2014) among individuals with reported access difficulties was higher in New England, West North Central, and Middle Atlantic, with a rate of 532, 469, and 425 per 10K cases, respectively. The rates are much lower between 2016 and 2017, with the highest rate reported for West South Central (78 per 10K presentations), as shown in Figure 6. The rate of presentation based on educational difficulties, as indicated by the ICD-9 inclusion criteria, showed no significant uptake. The West South Central and South Atlantic region showed the highest OUD rates among individuals with educational difficulties. This finding may be associated with specificity in the ICD 10 classification of SDOH.

## Discussion

This retrospective inpatient sample study shows the complexities and varied regional dynamics associated with inpatient OUD presentations across the country. The high rate and diversity of presentation by age group and sex show the complexity of targeting populations to mitigate the trend of presentation. The variance in age demographics has also been observed in other drug use and alcohol consumption studies [14-15]. The increasing rate by age group, Medicaid, and private insurance emphasizes the need for a providers' and payers' multifaceted approach to ensure practical continuous treatment approaches are available. The considerable rate of patients discharged against medical advice shown in this study could be directly related to the approach to care. The development of an effective outpatient approach and strategic social support tailored to individual dynamics could help mitigate the high cost of care in this patient population. The lack of resources and effective sustainability strategies for such approaches have impacted their success. Utilization of outpatient community models [16-17] that address some of the social factors contributing to presentations could be vital.

Racial dynamics are associated with several inequities that affect how we approach and understand health outcomes [18]. Studies have shown the disparities in health outcomes at various levels, including maternal health [19-20]. Given that rate increase has been observed among the white and Hispanic populations across the country as compared to other races with rate decrease, tailored educational materials could be vital in addressing the presentations' increase. The variance in presentation by race and demographics when comparing prescription opioid misuse and heroin use could provide some insights on the specific needs of each community when developing sustainable initiatives. A community-tailored approach to tackling such a problem is likely to be effective because of the continuous review of processes and data-driven strategies [21-22]. Given the high LOS, number of diagnoses (NDX), mean charges, severity at presentation, and significant access constraints among Native American populations [23-25], an outpatient community-based approach could be vital in mitigating the trends.

The social dynamics associated with equity in all communities are innately related to mental health and drug use and abuse in our communities. These socioeconomic factors, including access to healthcare service because of transportation or employment issues and quality education, play a role in these presentations, as demonstrated in this study. Education, employment, and other social factors are imperative for improving health outcomes among all populations in our communities [26]. Studies have found the intersection between unemployment, homelessness and drug use, abuse, and poor mental health in diverse communities [27-28]. This study provides further regional and demographics dynamics associated with OUD presentations across the United States. It also aids in reminding stakeholders of the potential fallout socioeconomic issues our societies are likely to be dealing with in the future, given the COVID-19 pandemic. Jefferies et al. show that unemployed individuals are likely to be at risk of depression and other mental health presentations, supporting our findings [29]. In recent years, federal and state legislation enacted to improve medical providers' continuing education, improving the quality and interoperability of local prescription drug monitoring programs, community recovery, drug disposal approaches, and community-based interprofessional collaboratives aimed at addressing the opioid epidemic are promising. They lay the groundwork in ensuring that communities have the necessary resources to provide appropriate and sustainable treatment approaches to the population they serve.

Family dynamics and support are an essential aspect of social life, health, and wellbeing. Populations such as adolescents and young adults are likely to be shaped by behaviors they observe or perceive as acceptable based on their family experiences. The differences and dynamics related to social and family support could help understand the high OUD presentation rate among young adults, as observed in this study. The support provided through community networks can aid in the improvement of health outcomes among these populations. The difficulties faced by individuals and communities are multifaceted and affect families in a variety of ways. Positive psychosocial dynamics can be related to positive health outcomes, especially among disadvantaged communities. As shown in this study, housing, among many other social factors, plays a significant role in the severity of adverse health outcomes. Such findings support the notion of a practical social component to medical treatment approaches, especially in drugs, addiction, and psychological care. Understanding and addressing these inequities in access and care based on psychosocial dynamics is imperative in improving communities' health across the United States [30]. Social support networks, active community engagement are essential in ensuring improve health outcomes. It is in the interest of all healthcare stakeholders to collaborate with policymakers to ensure the equitable allocation of resources to decrease individuals' likelihood of finding themselves in an abusive, violent, stressful, and discriminant environment, with no outlet and ability to improve their livelihood.

## Conclusions

This study shows that the social determinants of health play an essential role in OUD presentation across the country, particularly between low and high-income communities. The stigma often associated with OUD presentations often limits the patients' willingness for inpatient treatment, as demonstrated by the high discharge rate against medical advice in this study. Social justice dynamics need to be revisited to ensure effective allocation of resources to poor and low-income communities nationwide. The continuous need to create a more equitable society concerning resource allocation is clearly shown in this study as a necessity. The COVID-19 pandemic has exacerbated social factors (SDOH) such as employment and housing and disrupted support mechanisms in all communities across the country and will likely impact patient presentation in the coming years. This study's findings further aids in showing how imperative it is for public health officials, providers, and policymakers to work in tandem to improve health outcomes.

## Appendices

Appendix 1 

**Table 1:** ICD-9/10 Codes Inclusion Criteria ICD: International Classification of Diseases; OUD: Opioid Use Disorder

Description	ICD-9 Code	ICD-10 Code
Problems related to education and literacy	V62.3	Z55.XX
Problems related to employment and unemployment	V62.XX	Z56.XX
Occupational exposure to risk factors	V62.1	Z57.XX
Problems related to housing and economic circumstances	V60.XX	Z59.XX
Problems related to social environment	V62.89 V60.3 V62.4	Z60.XX
Problems related to upbringing	V61.XX	Z62.XX
Other problems related to primary support group, including family circumstances	V61.03 V61.49 V61.8 V61.9	Z63.XX
Problems related to certain psychosocial circumstances	V61.5 V61.6 V61.7 V62.81	Z64.XX
Problems related to other psychosocial circumstances	V62.5 V62.89 V62.9	Z65.XX
OUD INCLUSION CODES		
Opioid-Related Disorders	304.XX 305.XX	F11.XX

Appendix 2

**Table 2:** Sample characteristics and logistic regression analysis for OUD presentation among SDOH cases OUD: Opioid Use Disorder; SDOH: Social Determinant of Health; LOS: Length of Stay; NDX: Number of Diagnoses

Sample Characteristics						
Variables of Interest						
Inclusion (N-weighted)	2012-2014 (%)		2016-2017 (%)			
Combine SDOH Indicator	3002558(2.8)		1254899(1.8)			
Housing Difficulties	914315 (20)		687519 (45)			
Family Circumstances	356245 (12)		181070 (14)			
Psychosocial Issues	2113013 (55)		115655 (9)			
Healthcare Access	42005(1)		2190 (0.2)			
Unemployment	239625(8)		162580(8)			
Education & Literacy	32690(1)		19125(1.5)			
Upbringing			221860(10)			
Social Environment			160235(13)			
OUD Cases	367960(12)		153535(12)			
Logistic regression analysis for OUD presentation among SDOH cases (2012-2014 & 2016-2017)						
	2012-2014		2016-2017		P-value (Sig)	
Variables	OR	95 CI	OR	95 CI	2012-2014	2016-2017
Housing	0.91	0.90-0.92	1.49	1.47-1.52	<0.001	<0.001
Social Environment	0.71	0.55-0.58	0.85	0.82-0.87	<0.001	<0.001
Family Circumstances & Support	0.83	0.76-0.90	0.92	0.90-0.94	<0.001	<0.001
Psychosocial	0.71	0.70-0.72	0.90	0.86-0.94	<0.001	<0.001
Healthcare Access	1.72	1.62-1.83	0.54	0.44-0.67	<0.001	<0.001
Education & Literacy	0.81	0.76-0.85	0.43	0.39-0.47	<0.001	<0.001
Unemployment	1.16	1.15-1.18	1.37	1.34-1.39	<0.001	<0.001
Age Group (years)						
*<18					<0.001	<0.001
18-24	6.66	6.48-6.85	1.41	1.24-1.59	<0.001	<0.001
25-34	8.46	8.24-8.69	15.6	14.2-17.2	<0.001	<0.001
35-44	6.00	5.85-6.16	29.1	26.5-31.9	<0.001	<0.001
45-54	4.37	4.26-4.48	20.8	18.97-22.9	<0.001	<0.001
55-64	3.06	2.97-3.14	11.9	10.88-13.13	<0.001	<0.001
65+			5.73	5.20-6.32	<0.001	<0.001
Disposition						
Against Medical Advice	0.46	0.38-0.56	0.21	0.11-0.40	<0.001	<0.001
Died	1.77	1.47-2.15	0.75	0.39-1.4	<0.001	0.384
Transfer to Short-Term Hosp	0.93	0.77-1.12	0.34	0.18-0.64	0.467	0.001
Transfer Other: Includes Skilled Nursing Facility	0.98	0.81-1.19	0.40	0.21-0.76	0.890	0.005
Home Health Care (HHC)	0.83	0.68-1.00	0.38	0.20-0.72	0.05	0.003
*Routine						<0.001
LOS	1	0.99-1.01	0.99	0.99-.99	0.91	0.99
NDX	1.13	1.13-1.14	1.07	1.07-1.1	<0.001	<0.001
Sex						
Female	1.41	1.40-1.42	1.17	1.15-1.19	<0.001	<0.001
*Male						
Race						
*White						<0.001
Black	1.38	1.34-1.42	1.74	1.68-1.81	<0.001	<0.001
Hispanic	0.80	0.78-0.83	0.69	0.67-0.72	<0.001	<0.001
Asian or Pacific Islander	0.92	0.89-0.95	1.16	1.11-1.21	<0.001	<0.001
Native American	0.57	0.53-0.60	0.42	0.38-0.47	<0.001	<0.001
Other	1.35	1.27-1.44	1.22	1.13-1.32	<0.001	<0.001
Census Region						
*Northeast						<0.001
Middle Atlantic	1.78	1.75-1.82	2.04	1.98-2.10	<0.001	<0.001
East North Central	1.70	1.71-1.77	1.94	1.89-1.99	<0.001	<0.001
West North Central	1.03	0.99-1.03	1.17	1.14-1.20	0.126	<0.001
South Atlantic	0.95	0.93-0.97	064	0.61-0.66	<0.001	<0.001
East South Central	1.08	1.06-1.10	1.15	1.12-1.18	<0.001	<0.001
West South Central	1.38	1.35-1.41	1.30	1.26-1.35	<0.001	<0.001
Mountain	0.76	0.75-0.78	0.48	0.46-0.50	<0.001	<0.001
Pacific	0.91	0.88-0.92	0.88	0.85-0.91	<0.001	<0.001
Primary Payer						
Medicare						<0.001
Medicaid	0.72	0.69-0.72	0.77	0.74-0.85	<0.001	<0.001
Private Insurance	1.01	0.99-1.02	1.20	1.16-1.24	0.221	<0.001
Self-Pay	0.78	0.76-0.79	0.80	0.77-0.83	<0.001	<0.001
No Charge	1.14	1.12-1.16	0.84	0.81-0.87	<0.001	<0.001
Other	1.30	1.26-1.34	0.78	0.72-0.83	<0.001	<0.001
Rural Urban Code						
*"Central" counties of metro areas of >=1 million population						<0.001
"Fringe" counties of metro areas of >=1 million population	1.25	1.21-1.27	1.62	1.56-1.68	<0.001	<0.001
Counties in metro areas of 250,000-999,999 population	1.15	1.12-1.18	1.52	1.46-1.57	<0.001	<0.001
Counties in metro areas of 50,000-249,999 population	1.12	1.1-1.15	1.21	1.17-1.25	<0.001	<0.001
Micropolitan counties	0.95	0.93-0.98	1.11	1.06-1.15	<0.001	<0.001
Not metropolitan or micropolitan counties	1.06	1.01-1.10	0.87	0.84-0.89	<0.001	0.005
Median Household Income						
*0-25th percentile					<0.001	
26th to 50th percentile (median)	0.97	0.96-0.98	1.02	0.99-1.03	<0.001	0.085
51st to 75th percentile	0.88	0.87-0.89	0.94	0.92-0.96	<0.001	<0.001
76th to 100th percentile	0.98	0.96-0.99	0.96	0.95-0.98	<0.001	0.046
Severity Risk						
*No class specified						
Minor loss of function (includes cases with no comorbidity or complications)	0.955	0.92-0.98	0.61	0.56-0.59	0.008	<0.001
Moderate loss of function	0.96	0.92-0.99	0.64	0.60-0.67	<0.001	<0.001
Major loss of function	1.21	1.17-1.25	0.79	0.75-0.84	<0.001	<0.001
Extreme loss of function	1.08	1.05-1.12	0.89	0.85-0.94	<0.001	<0.001
Mortality Risk (%)						
*No class specified						
Minor likelihood of dying	2.20	1.85-2.62	1.05	0.60-1.83	<0.001	0.85
Moderate likelihood of dying	1.17	1.13-1.22	1.55	1.46-1.64	<0.001	<0.001
Major likelihood of dying	0.85	0.82-0.88	0.89	0.85-0.94	<0.001	<0.001
Extreme likelihood of dying	0.86	0.83-0.89	0.77	0.73-0.81	<0.001	<0.001
